# Structure of the Membrane Anchor of Pestivirus Glycoprotein E^rns^, a Long Tilted Amphipathic Helix

**DOI:** 10.1371/journal.ppat.1003973

**Published:** 2014-02-27

**Authors:** Daniel Aberle, Claudia Muhle-Goll, Jochen Bürck, Moritz Wolf, Sabine Reißer, Burkhard Luy, Wolfgang Wenzel, Anne S. Ulrich, Gregor Meyers

**Affiliations:** 1 Institut für Immunologie, Friedrich-Loeffler-Institut, Greifswald – Insel Riems, Germany; 2 Karlsruhe Institute of Technology, Institut für Organische Chemie, Karlsruhe, Germany; 3 Karlsruhe Institute of Technology, Institut für Biologische Grenzflächen (IBG-2), Karlsruhe, Germany; 4 Karlsruhe Institute of Technology, Institut für Nanotechnologie, Karlsruhe, Germany; Institut Pasteur, France

## Abstract

E^rns^ is an essential virion glycoprotein with RNase activity that suppresses host cellular innate immune responses upon being partially secreted from the infected cells. Its unusual C-terminus plays multiple roles, as the amphiphilic helix acts as a membrane anchor, as a signal peptidase cleavage site, and as a retention/secretion signal. We analyzed the structure and membrane binding properties of this sequence to gain a better understanding of the underlying mechanisms. CD spectroscopy in different setups, as well as Monte Carlo and molecular dynamics simulations confirmed the helical folding and showed that the helix is accommodated in the amphiphilic region of the lipid bilayer with a slight tilt rather than lying parallel to the surface. This model was confirmed by NMR analyses that also identified a central stretch of 15 residues within the helix that is fully shielded from the aqueous layer, which is C-terminally followed by a putative hairpin structure. These findings explain the strong membrane binding of the protein and provide clues to establishing the E^rns^ membrane contact, processing and secretion.

## Author Summary

The E^rns^ protein (envelope protein, RNase, secreted) of pestiviruses represents one of the most fascinating proteins in virology. E^rns^ is not only an essential structural component of the virus particle but also an unspecific RNase. The latter activity is dispensable for pestivirus replication but represents a virulence factor involved in the establishment of lifelong persistent infection. These functions of E^rns^ are connected with its repressive activity on the type I interferon response of the infected host probably depending on secretion of part of the protein synthesized within the infected cell followed by its distribution with the blood stream. To understand the mechanisms leading to an equilibrium between intracellular retention (for production of virus particles) and secretion (for repression of the innate immune response) the principles of E^rns^ membrane binding need to be better understood. The recently published E^rns^ crystal structure, however, is lacking the relevant carboxyterminal membrane anchor region. We report here structure analyses of the E^rns^ membrane anchor bound to model membranes. This work was based on circular dichroism, nuclear magnetic resonance spectroscopy, and structure simulations, and revealed a new type of membrane anchor for a surface protein. These data will help to explain the unusual functions of E^rns^.

## Introduction

The genus *Pestivirus* belongs to the family *Flaviviridae*, together with the genera *Hepacivirus*, *Flavivirus* and *Pegivirus*. It represents a group of economically important animal viruses like classical swine fever virus (CSFV) and bovine viral diarrhea virus (BVDV) [1]. The economical damage caused by these viruses is not only due to the acute infection of the animals, but stems also from their ability to cause persistent infection of the fetus after infection of a pregnant animal [2], [3]. If an infection of the fetus by BVDV is established, a persistently infected calf is born. This calf often shows no signs of disease, but sheds huge amounts of infectious virus particles throughout its whole life, which leads to an efficient spreading of the virus.

The genome organisation and basic molecular features of pestiviruses are more similar to the hepacivirus HCV (human hepatitis C virus) than to the other members of the *Flaviviridae*. The positive sense single stranded RNA genome consists of about 12,300 nucleotides and contains one single open reading frame that codes for a single polyprotein precursor of ∼4000 amino acids [1]. This precursor is co- and posttranslationally processed by cellular and viral proteases to release the viral proteins. Compared to the HCV RNA, the pestivirus genome codes for two additional proteins: the non-structural protein N^pro^, and the structural protein E^rns^ [1]. Both proteins interfere with the immune response of the infected animal and are important for establishing a persistent infection [4]. The two proteins exert two different functions. N^pro^ is an autoprotease and leads to the degradation of IRF3 (interferon regulatory factor 3) in the infected cell via the proteasome [5], [6], [7], [8], [9], [10], and it also interferes with IRF7 dependent pathways [11]. This results in the deactivation of the innate immune response of the infected cell. In contrast, E^rns^ is a viral glycoprotein that forms disulfide-linked homodimers and can be found together with the glycoproteins E1 and E2 on the surface of the enveloped virus particle [3], [12], [13]. E^rns^ consists of 227 amino acids, has a molecular weight of 42–48 kDa, being heavily glycosylated except for its C-terminal region [3], [14], [15]. It is not only involved in the formation of infectious virus particles, but it is also secreted from the infected cells, and up to 50 ng/ml of the protein can be detected in the blood of infected animals [16]. In addition, E^rns^ has an intrinsic RNase activity, which is very unusual for an RNA virus protein [17], [18], [19]. The structure of the RNase domain of E^rns^ was recently determined, with the finding that it has a T2-RNase like fold [20] that confirmed data from sequence analysis studies [18]. T2-RNase represents a very old and unique RNase family whose members are broadly distributed in nature, but the functions of these RNases are unknown. The enzymatic activity of E^rns^ is necessary for its activity as a virulence factor. It could be shown that the deactivation of the RNase activity by deletion of one amino acid in the active site of the protein caused attenuation of the virus in its natural host [21], [22].

Interestingly, attenuation was also observed in an RNase positive virus, in which E^rns^ dimerization was prevented by mutation of the Cys residue that forms the intermolecular disulfide bond in the wt protein [23]. Moreover, an abrogation of the E^rns^ RNase activity together with a deletion of the N^pro^ coding sequence prevented the establishment of a persistent BVDV infection [4]. We and others postulated that the secreted version of E^rns^ - and not the protein bound to the virus particle - represents the key player that interferes with the immune system. The secretion of E^rns^ is controlled by the C-terminal end of the protein. In previous studies we have determined several parameters in the C-terminal region of E^rns^ that are necessary for the retention/secretion of the protein [24], [25], [26]. The free C-terminus of E^rns^ is formed upon cleavage of the E^rns^/E1 precursor protein by the cellular signal peptidase [27]. This cleavage site is a very unusual substrate for the signal peptidase, because the characteristic signal activating this peptidase is normally composed of a transmembrane helix followed by a so-called von Heijne sequence [28], [29]. The cleavage site between E^rns^ and E1 contains such a von Heijne sequence, but the E^rns^ C-terminus lacks a transmembrane helix and contains an amphipathic helix instead. Nevertheless, the C-terminus of E^rns^ obviously has to fold in a certain conformation that is accepted as a substrate by the signal peptidase.

**Figure 1:**
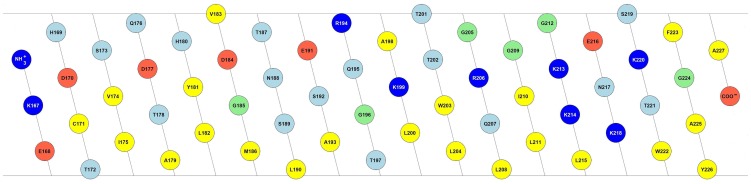
Amphipathic helix model of the E^rns^ membrane anchor. 2D flat projection of the 3D structure of the E^rns^ anchor (Lys167 – Ala227) assuming a continuous α-helical conformation. Positively charged amino acids are shown in dark blue (Arg, Lys), negatively charged ones in red (Asp, Glu), and hydrophobic amino acids are colored in yellow (Leu, Val, Ile, Met, Trp, Tyr, Phe, Ala, Cys). Polar amino acids are displayed in light blue (Thr, Asn, Ser, Gln, His), and the remaining ones (Gly, Pro) in green. The illustration was generated with the in-house software “Protein Origami” (Karlsruhe Institute of Technology, http://www.ibg.kit.edu/nmr/544.php).

The C-terminus of E^rns^ governs not only protein cleavage and secretion, but it is also important for the membrane binding of the protein. Sequence analysis predicts a helical fold for the C-terminus, which would bestow it with a marked amphipathic character [24], [25]. **Fig. 1** shows the 2D flat projection of the 3D structure of the E^rns^ anchor (Lys167 – Ala227) assuming a continuous α-helical conformation. The hydrophilic and hydrophobic faces are maintained throughout the entire length of the helix, which implies that it could bind flat onto the membrane surface. This amphipathic structure would explain the membrane anchoring, but not the action of the signal peptidase, nor the regulation of secretion. As an alternative arrangement, it has been recently suggested that the E^rns^ membrane anchor might fold as a helical hairpin by forming a long ladder of salt bridges between its N-terminal and C-terminal helical segments, a so-called electrostatic ‘charge zipper’ [30]. The resulting amphiphilic hairpin would in principle have an appropriate length to span the lipid bilayer in a transmembrane alignment and could thus serve as a substrate for the signal peptidase.

To better understand the role of E^rns^ and its mechanism of membrane anchoring and secretion, we have recently obtained some initial data on short peptide fragments from this region of the protein when bound to lipid bilayers [26], but the non-continuous nature of these sequences precluded any firm interpretation. Here, we show that the complete C-terminal anchor of E^rns^ indeed adopts a continuous α-helical fold when bound to the membrane. In contrast to the simplistic pictures of a surface-bound helix or a transmembrane hairpin, however, our results show that the E^rns^ C-terminus is slightly tilted with regard to the membrane surface, and a substantial stretch of residues is shielded from the aqueous phase by the hydrophobic environment, while the rest is located at the water/membrane interphase. This refined model represents the first example of a viral structural protein that is bound to a membrane via an amphipathic helix.

## Results

### Secondary structure of the E^rns^ anchor

**Figure 2:**
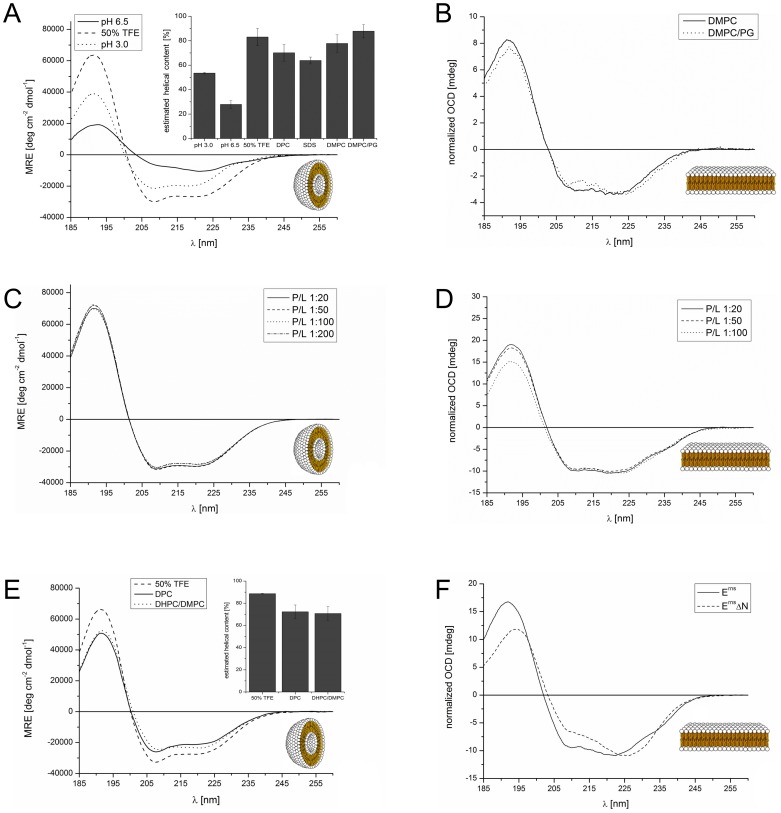
CD and OCD spectra of the E^rns^ C-terminus and the N-terminally truncated version E^rns^ΔN in different environments. (A) CD spectra of the E^rns^ membrane anchor (Lys167 – Ala227) in phosphate buffer at pH 6.5 (straight line), at pH 3 (dotted line), or in 50% TFE at pH 6.5 (dashed line). The results of the secondary structure analysis in these environments as well as in detergent micelles and lipoid vesicles are displayed in the inserted bar graph. The bars represent the mean helix content of the E^rns^ anchor calculated with three secondary structure calculation programs (CDSSTR, CONTIN-LL and SELCON-3). (B) OCD spectra of the E^rns^ membrane anchor (Lys167 – Ala227) in oriented lipid bilayers composed of DMPC (straight line), or a mixture of DMPC/DMPG (1∶1) (dotted line), each with a protein/lipid ratio of 1∶100. The spectra were normalized to the same intensity at ∼220 nm to illustrate the similarity in the lineshapes. (C) CD spectra of the E^rns^ membrane anchor (Lys167 – Ala227) in DMPC/DMPG (1∶1) vesicles, recorded at protein/lipid ratios of 1∶20 (straight line), 1∶50 (dashed line), 1∶100 (dotted line), and 1∶200 (dashed-dotted line). (D) OCD spectra of the E^rns^ membrane anchor (Lys167 – Ala227) in oriented lipid bilayers composed of a mixture of DMPC/DMPG (1∶1). The spectra were recorded at protein/lipid ratios of 1∶20 (straight line), 1∶50 (dashed line), and 1∶100 (dotted line). The spectra were normalized to the same intensity at ∼220 nm to illustrate the similarity in the lineshapes. (E) CD spectra of N-terminally truncated E^rns^ΔN (Arg194 – Ala227) in 50% TFE (dashed line), 10 mM DPC micelles (straight line), and bicelles composed of DHPC/DMPC (4∶1) (dotted line), at a protein/lipid ratio of 1∶100. The results of the secondary structure analysis in these environments are displayed in the inserted bar diagrams. The bars represent the mean helix content of E^rns^ΔN estimated with three secondary structure calculation programs (CDSSTR, CONTIN-LL and SELCON-3). (F) Comparison of the OCD spectra of the E^rns^ membrane anchor (Lys167 – Ala227) and the N-terminally truncated E^rns^ΔN (Arg194 – Ala227) in oriented lipid bilayers composed of a mixture of DMPC/DMPG (1∶1) at a protein/lipid ratio of 1∶50. The spectra were normalized to the same intensity at ∼220 nm to allow for a better comparison of the lineshapes.

In a previous analysis we had examined three overlapping peptide fragments corresponding to the E^rns^ anchor sequences of CSFV strain Alfort/Tübingen [26] and BVDV strain CP7 (data not shown). Their CD analysis revealed a strong tendency for the middle and the C-terminal part of the anchor sequence, represented by the two corresponding peptides, to fold as a helix. To verify these results for the entire E^rns^ anchor (Lys167 – Ala227), we determined the secondary structure of this 61-residue domain in different environments by CD, as shown in **Fig. 2**. We first used a solution of 50% TFE in phosphate buffer (PB) pH 6.5 (**Fig. 2 A**, **dashed line**), which promotes intramolecular hydrogen bonds. This CD spectrum of the E^rns^ anchor showed an overall helical fold, and the secondary structure deconvolution revealed a helix content of 80% for these data (**Fig. 2 A**, **bar diagram**). Thus, almost the complete E^rns^ C-terminus is in principle able to adopt a helical conformation. To test whether this secondary structure is also present in pure phosphate buffer (PB), we measured the E^rns^ anchor in PB pH 6.5 (**Fig. 2 A**, **solid line**) and pH 3 (**Fig. 2 A**, **dotted line**). However, the E^rns^ anchor was only partially soluble at pH 6.5, leading to protein aggregation and turbidity in the aqueous sample. Therefore, the CD lineshape shows spectral artefacts caused by absorption flattening and differential scattering at wavelengths <215 nm, and the secondary structure analysis yielded only a very low degree of helix content. At pH 3, on the other hand, the protein is completely soluble in PB, and the secondary structure calculation revealed a helix content of about 50%.

To analyse the secondary structure of the E^rns^ anchor in a membrane-like environment, we first used detergent micelles in low salt buffer. As the E^rns^ anchor is positively charged we compared micelles of zwitterionic DPC and negatively charged SDS. Both systems supported a strong helical folding, and the secondary structure deconvolution revealed a helical fraction of over 60% (**Fig. 2 A**, **bar diagram**). We then used zwitterionic DMPC lipid vesicles, and a 1∶1 mixture of DMPC with anionic DMPG. Both systems induced a high degree of helical folding (∼70–80%) (**Fig. 2 A**, **bar diagram**), similar to 50% TFE. These results indicate that the E^rns^ C-terminus has a helix content of up to 80% in a membrane (-mimicking) environment, and may therefore be present as a continuous amphipathic helix, as implicated in **Fig. 1**.

### Orientation of the E^rns^ amphipathic helix relative to the membrane surface

To examine the orientation of the helical sequence relative to the membrane surface we used oriented CD (OCD). In macroscopically oriented membrane samples it is possible to estimate the tilt angle of a membrane bound helix from the intensity of the negative band at 208 nm in the OCD spectrum, which is polarized parallel to the helix axis [31], [32], [33]. If the helix adopts an alignment parallel to the membrane surface, as expected for E^rns^, the minimum at 208 nm has a stronger intensity than the minimum around 223 nm. In contrast, a transmembrane helix that lies perpendicularly to the membrane surface shows no negative band at 208 nm, or even some positive ellipticity (Supporting Information Fig. S1). The OCD spectra of the E^rns^ anchor, recorded in oriented DMPC or DMPC/DMPG (1∶1) (**Fig. 2 B**), show a pronounced minimum at 208 nm with nearly the same intensity as the one at 223 nm. A transmembrane alignment of the E^rns^ anchor, as had been speculated for the E^rns^/E1 precursor [30], can thus be excluded for the mature processed E^rns^ under these conditions. However, because the intensity of the 208 nm minimum is less than the intensity of the 223 nm band, the helix does not seem to be aligned completely parallel to the membrane surface. Without taking any minor secondary structure elements into account (e.g. disordered regions, which have a minimum at 198 nm), the E^rns^ anchor appears to be slightly tilted within the membrane.

### Orientation of the E^rns^ anchor is independent of concentration

Many amphipathic helices, especially antimicrobial peptides, are known to interact with each other in a concentration dependent manner, which often results in changes in their membrane alignment. At low concentrations these peptides exhibit a mostly parallel orientation with regard to the membrane surface, but at higher concentrations the self-assembly of these molecules leads to a re-alignment and the formation of a transmembrane pore [33], [34], [35], [36], [37], [38]. In the case of E^rns^, the possibility of self-assembly via an intermolecular charge zipper had been suggested [30], or helix-helix interactions via a GxxxG motif might be conceivable. To test for a putative concentration-dependent change in the secondary structure (e.g. aggregation or oligomerization) or in the orientation of the helical segment of E^rns^, we compared several samples with different lipid/protein ratios. The CD spectra of all four tested concentrations (P/L ratios) in the DMPC/DMPG 1∶1 lipid mixture were essentially identical (**Fig. 2C**). Neither did the OCD spectra at the same ratios of 1∶20, 1∶50 and 1∶100, as displayed in **Fig. 2D**, show any change in the alignment of the helical segment. (The OCD sample at 1∶200 did not yield a reliable spectrum due to technical problems resulting from the very low protein concentration.) In summary, these CD and OCD data demonstrate that the E^rns^ anchor does not show a concentration-dependent change in its global secondary structure nor in its orientation in the membrane. Most importantly, the data recorded here give no indication at all of a transmembrane alignment at any concentration.

### Secondary structure of the far C-terminal region of E^rns^

The observed peripheral location of the C-terminal part of the E^rns^ membrane anchor is highly intriguing, because this architecture represents a so far unknown type of substrate for the cellular signal peptidase, which usually requires a transmembrane helix upstream of the cleavage site. To investigate the structure of the C-terminal fragment in more detail, we expressed a truncated construct named E^rns^ΔN, which represents the far C-terminal 34 amino acids of the anchor sequence (Arg194 – Ala227). To analyse the secondary structure of this C-terminal E^rns^ anchor fragment (**Fig. 2 E**), we used 50% TFE (dashed line), DPC micelles (straight line), and DMPC vesicles (not shown). Unfortunately, the CD spectrum of E^rns^ΔN in DMPC vesicles showed scattering artefacts which lead to an error-prone CD spectrum and prevented an exact analysis. To allow comparison with the subsequent NMR analysis, we examined the C-terminal region also in small lipid bicelles with DHPC/DMPC (4∶1) (dotted line). The corresponding CD spectra of E^rns^ΔN showed a high percentage of helical folding (∼70–90%) in all three systems (**Fig. 2 E**, **bar diagram**). This means that the E^rns^ anchor can fold as a long amphipathic helix not only in detergent micelles and lipid vesicle suspensions, but also in bicelles.

The OCD spectrum of the C-terminal fragment in **Fig. 2F** shows a less intensive band at 208 nm than the complete E^rns^ anchor at a P/L ratio of 1∶50. This means that either the N-terminal region of the E^rns^ anchor is less tilted in the membrane than the C-terminal region, or the N-terminal elongation pulls the C-terminal region into a more parallel orientation. Importantly, the band at 208 nm still has a distinct negative signal amplitude, which excludes the possibility that this region of the E^rns^ anchor could be inserted into the membrane in a transmembrane alignment.

### Detailed NMR structure analysis of the far C-terminal region of the E^rns^ anchor

Further information on the orientation of the E^rns^ anchor in the membrane, the positions of individual residues, and the water accessibility of individual NH groups was obtained from liquid-state NMR spectroscopy. Although the full-length E^rns^ anchor yielded good quality ^1^H^15^N-HSQC spectra in DHPC/DMPC bicelles, as well as in DPC and SDS micelles, the corresponding 3D-^15^N-HSQC-NOESY and TOCSY spectra suffered from line broadening that impeded sequential assignment (Supporting Information Figure S2). The stretch Trp203-Gly212 could be tentatively assigned based on characteristic proton shifts, but the assignment could only be safely confirmed later by comparison with the spectra of E^rns^ΔN. Therefore, the detailed NMR analysis was done for the truncated E^rns^ΔN corresponding to the 34 C-terminal residues of E^rns^. Backbone assignment (^1^H, ^15^N, ^13^Cα, ^13^Cβ) was achieved for all residues, except for the first two in DHPC/DMPC (4∶1) bicelles.

We sought to determine whether parts of E^rns^ΔN are protected from the solvent, by titration with the paramagnetic agent Gd-DOTA and by dissolution of a lyophilized sample of E^rns^ΔN/DHPC/DMPC in D_2_O. Supporting Information Figure S3 shows that residue protection factors of E^rns^ΔN with Gd DOTA at 0.5 mM are uniform along the entire sequence. D_2_O exchange revealed no stably protected residues either (data not shown), given that already a single exchange event leads to a disappearance of the signal. Similarly, titration with paramagnetic agents shows a protection only when the residues are deeply inserted in the hydrophobic interior of the bicelle [39]. We therefore determined also the water accessibility of the NH groups with the more sensitive CLEANEX-PM pulse sequence [40], which measures proton exchange rates. The CLEANEX pulse sequence applies a water-selective excitation pulse prior to a chemical exchange sequence in which magnetization transfer from water protons to ^15^N-bound exchangeable protons occurs, followed by a ^1^H^15^N-HSQC-type experiment. The chemical exchange efficiency depends on the accessibility of the ^15^N-bound proton, and thus reports on its location in the bicelle or its stable participation in a hydrogen bond. Here, a single encounter with a water molecule may already lead to a measurable magnetization, meaning that for partially solvent-accessible amide protons this method is more sensitive than the other two, because the protein exchange time is limited to 100 ms.

**Figure 3:**
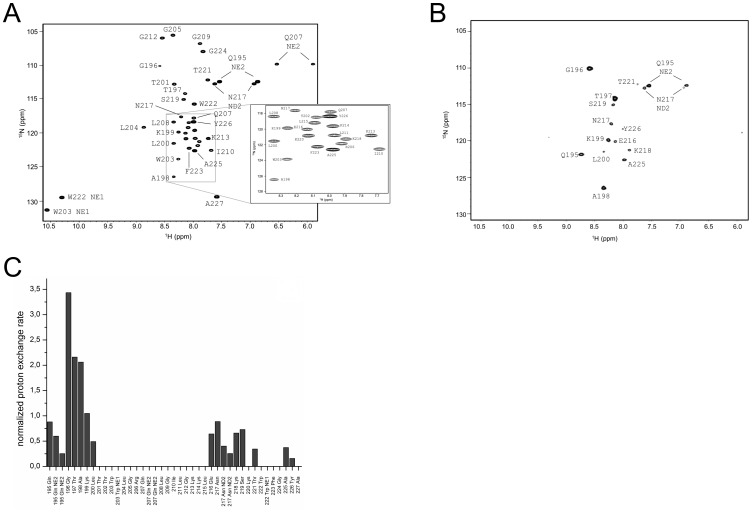
Water accessibility measurement of E^rns^ΔN. (A) ^15^N-HSQC, and (B) CLEANEX spectra of N-terminally truncated E^rns^ΔN (Arg194 – Ala227) in bicelles composed of DHPC/DMPC (4∶1) at a protein/lipid ratio of 1∶222. NH cross peaks are labelled with the name of the amino acid in single letter code and its number according to the full-length E^rns^ protein. The central part of the spectrum in (A) is blown up in the box on the right for better clarity. The side chain NH groups of Trp203 and 222 are marked as NE1. The Gln207 side chain, together with the side chains of Gln195 and Asn217 could not be further assigned and are therefore marked as NE2 or ND2. (C) Calculated normalized proton exchange rates of all identified NH groups in the spectra. The NH groups are labelled as in the NMR spectra.

**Figure 4:**
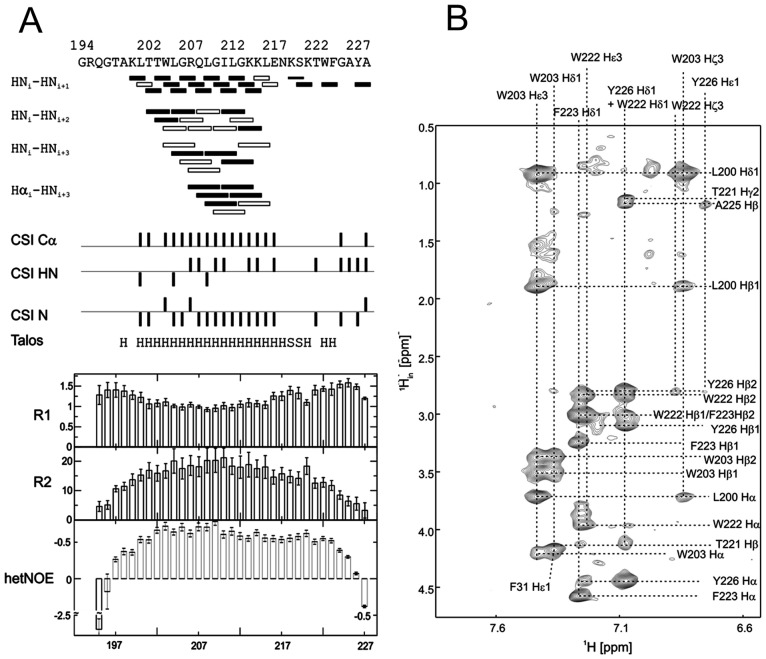
Secondary structure prediction of E^rns^ΔN deduced from NMR spectroscopy. (A) The measured HN-NH contact is displayed for each amino acid of E^rns^ΔN (Arg194 – Ala227) in the upper part of the figure to predict the secondary structure of the region. The CSI (chemical shift index) of Cα, HN and N determined by triple-resonance experiments for the protein backbone are shown for each amino acid in the middle part of the figure together with the resulting calculated secondary structure of TALOS (Torsion Angle Likelihood Obtained from Shift and sequence similarity) for each amino acid. The measured ^15^N longitudinal (R1) and transversal (R2) relaxation rates are displayed in the lower part of the figure as well as the heteronuclear NOE (het-NOE). (B) ^1^H-^1^H contacts of the C-terminal end of E^rns^ΔN (Arg194 – Ala227) that reveal steric proximity. The identified protons are labelled by their IUPAC nomenclature and by the corresponding amino acid and numbered according to the full-length E^rns^ protein.

The assigned ^1^H^15^N-HSQC of E^rns^ΔN is shown in **Fig. 3A**. Judging from the nuclear Overhauser effect (NOE) and chemical shift anisotropy (CSI) patterns (**Fig. 4A** **, middle part**), the helix extends from Leu215 or Glu216, corresponding to 50% helical content. These findings support the CD data above, that showed a high degree of helical folding in the same membrane-mimetic environment (data above). The remaining residues in the C-terminus show some HN-HN contacts as well as NOEs between Trp222, Phe223 and Tyr226 (Fig. **4B**), which suggests a loop-like structure, but the quality of the spectra did not yield enough NOEs for a full 3D structure determination. Nonetheless, the HN-HN contacts together with the loop-like contacts in the C-terminus (**Fig. 4A** **, upper part**) and the water accessibility measurements argue in favour of a dynamic loop-like conformation of the far C-terminus rather than a firmly folded α-helical structure.

A comparison of the ^15^N-HSQC and the CLEANEX spectra (**Fig. 3B**) revealed that only 13 amide protons are engaged in exchange (i.e. they give cross-peaks in the assigned CLEANEX spectrum). We may thus conclude that the remaining 20 amide groups are protected from exchange and are therefore assume a stable secondary structure within a more hydrophobic environment characterized by a low dielectric constant. A comparison with the water-HN NOEs in a ^1^H^15^N-NOESY experiment confirmed our analysis. To obtain the water accessibility of each NH group, we calculated the normalized proton exchange rate by dividing the intensity of each CLEANEX peak by the intensity of the corresponding ^1^H-^15^N HSQC peak. **Fig. 3C** shows that a long stretch of 15 amino acids in the middle of E^rns^ΔN does not have any water contact at all and should therefore be protected within the bicelle. This stretch comprises not only hydrophobic amino acids, but also contains two Thr, one Arg, two Lys and one Gln. Most remarkably, the side chains of Gln207 and Trp203, which are positioned on the edge of the hydrophobic face of the helix, are not in contact with water either, thus again supporting the model of an amphiphilic helix that is embedded in and protected by the membrane. In contrast, the N-terminal region of E^rns^ΔN up to Leu200 shows a high normalized proton exchange rate that is characteristic of a water-exposed surface-location. Surprisingly, most of the C-terminal residues, including the very last amino acid Ala227, are again shielded from water.

^15^N-NMR relaxation analysis (**Fig. 4A** **, lower part**) suggests that the residues comprising the helical part are fairly rigid. The T1, T2 and hetNOE values show a tendency to gradually change from residue 201 towards the N-terminus and from residue 215 or 216 towards the C-terminus, indicating increasing flexibility towards the ends. Besides the immediate N-terminus up to residue 196 (in accordance with the lack of data for the first residues), the last two residues of the C-terminus are highly flexible, which is evident from their high T2 values and low to negative hetNOEs. Together with the fact that these two and the preceding residues at the utmost C-terminal end are protected from contact with water and show an interconnecting network of NOEs, a picture emerges where the C-terminus forms a stable but semi-flexible structure within the bicelle, whereas the N-terminus is unstructured and exposed on the surface of the bicelle.

### Structural influence of the C-terminus

**Figure 5:**
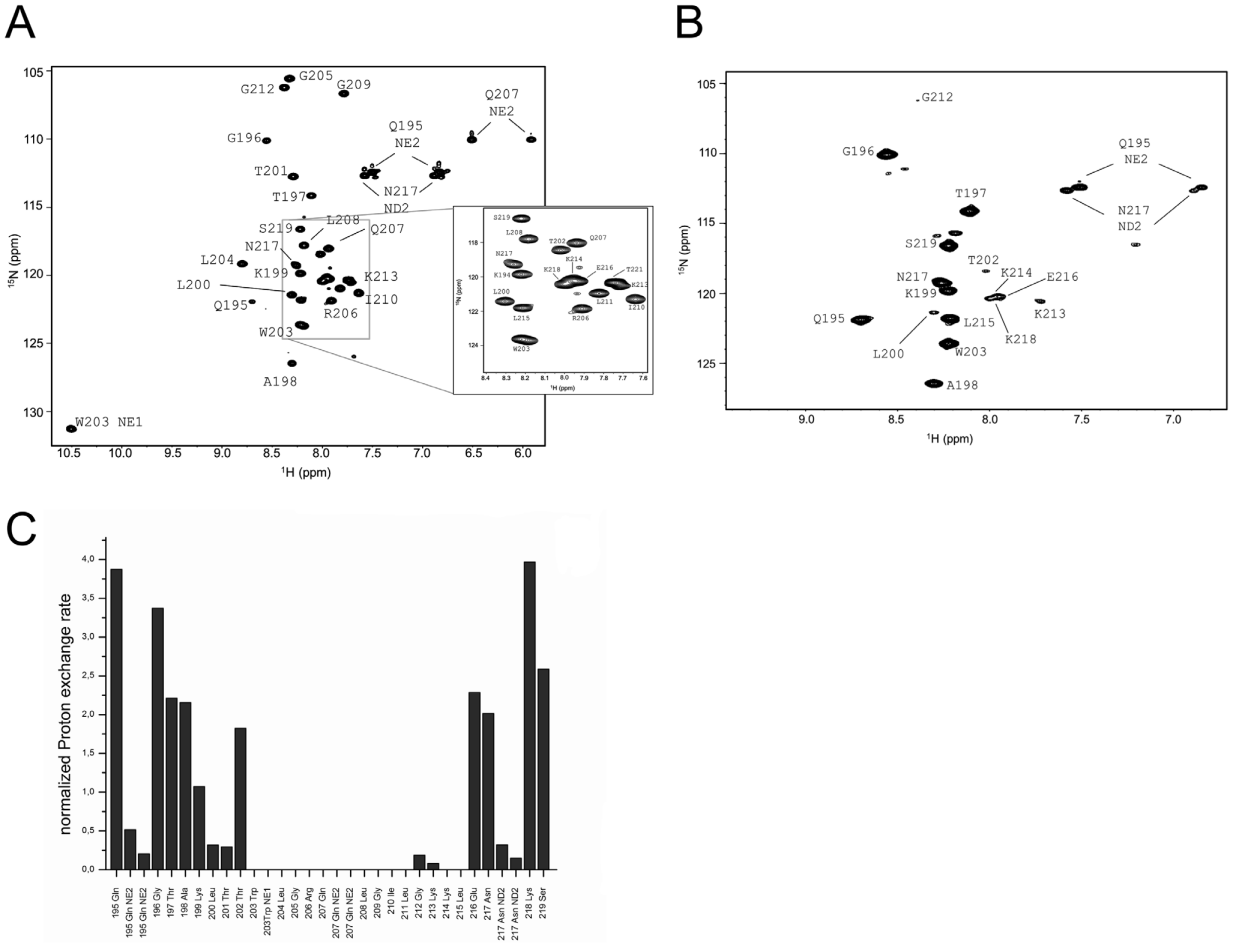
Water accessibility measurements of E^rns^ΔNΔC. (A) ^15^N-HSQC, and (B) CLEANEX spectra of E^rns^ΔNΔC (Arg194 – Thr221), a 6 amino acid C-terminal truncated version of E^rns^ΔN, recorded in a bicelle system composed of DHPC/DMPC (4∶1) and a protein/lipid ratio of 1∶222. The ^15^N chemical shift is displayed on the y-axis and the ^1^H shift is shown on the x-axis. Identified NH groups are marked with the single letter code of the corresponding amino acids and numbered according to the full-length E^rns^ protein. The central spectrum area in (A) is shown as a blow up in the box on the right for better clarity. The side chain NH groups of Trp203 and 222 are marked as NE1. The Gln207 side chain, together with the side chains of Gln195 and Asn217 could not be further assigned and are therefore marked as NE2 or ND2. (C) Calculated normalized proton exchange rates of all identified NH groups of the spectra. Lys220 and Thr221 could not be assigned unambiguously and therefore are not presented in the figure.

Earlier cell culture studies had reported that the C-terminus of E^rns^ (BVDV Strain CP7) has a major influence on the retention/secretion of the protein. The deletion of the last five C-terminal amino acids (FGAYA) led to a dramatic increase in the amount of secreted E^rns^ [24]. A recently conducted cell culture experiment of the E^rns^ protein from CSFV strain Alford/Tübingen showed that even the loss of the four C-terminal residues (GAYA) has a major impact on protein secretion. In these experiments the protein secretion rate increased from below 10% (wt protein) to 35% (truncated version) (unpublished data). Since the major part of the membrane binding of the E^rns^ anchor is supposedly contributed by the central region of the amphipathic helix, we wondered whether the C-terminus may have any influence on the conformation of the membrane anchor, thereby modulating its membrane association. To answer this question, we expressed and analysed yet another, C-terminally trunctated protein E^rns^ΔNΔC, a variant of E^rns^ΔN lacking the last 6 amino acids (WFGAYA). The assignment of E^rns^ΔN could be easily transferred to the ^1^H-^15^N HSQC spectrum of this shortened version (**Fig. 5A**). The corresponding CLEANEX spectrum (**Fig. 5B**) and the calculated normalized proton exchange rates (**Fig. 5C**) indicated that in E^rns^ΔNΔC only 8 amino acids located in the middle of the sequence were shielded from water. In addition, two further amino acids, Thr201 on the N-terminal side and Lys214 on the C-terminal side of the shielded sequence showed no water contact.

**Figure 6:**
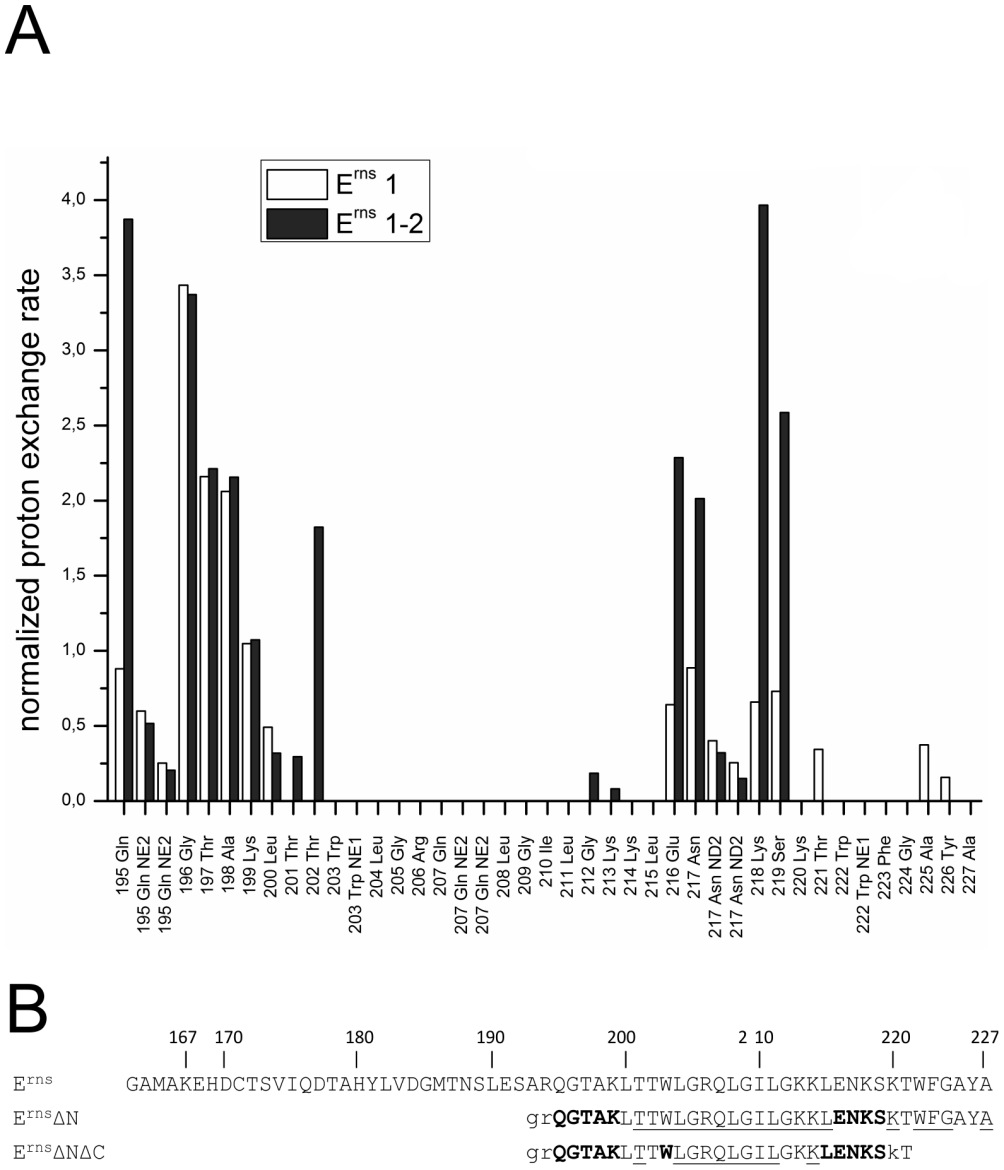
Comparison of the proton exchange rates of E^rns^ΔN and E^rns^ΔNΔC. (A) Normalized proton exchange rates of E^rns^ΔN (Arg194 – Ala227) and of E^rns^ΔNΔC (Arg194 – Thr221) for all NH groups identified in the spectra (see Fig. 3C and 5C). (B) The amino acid sequences of the E^rns^ membrane anchor (E^rns^), the N-terminally truncated protein E^rns^ΔN, and the N- and C-terminally truncated E^rns^ΔNΔC are numbered according to the full-length E^rns^ protein. The amino acids of E^rns^ΔN and E^rns^ΔNΔC are displayed in different fonts depending on the value of the proton exchange rate of the corresponding peptide NH group. NH groups with a proton exchange rate above of 0.5 are shown in bold face, while those with a proton exchange rate below 0.5 are in standard font. The underlined amino acids were not detected in the CLEANEX spectrum and therefore do not show any significant proton exchange. Unassigned amino acids in the ^15^N-HSQC spectrum leading to a general lack of information about their water accessibility are presented in lower case.

Comparison of the normalized proton exchange rates of E^rns^ΔN and E^rns^ΔNΔC in **Fig. 6A** reveals several differences resulting from the deletion of the 6 C-terminal amino acids. For the N-terminal amino acids, E^rns^ΔN and E^rns^ΔNΔC yielded nearly the same values (except for the peptide bond NH group of Gln195), hence the conformation and water-exposed location of these residues should be equivalent in both proteins. However, residues Thr201, Gly212 and Lys213, which in the case of E^rns^ΔN are embedded in the bicelle, now seem to be located somewhat closer to the lipid/water interphase in E^rns^ΔNΔC, as seen by the small but significant change in the proton exchange rate. Especially the peptide bond of Trp203 shows a high proton exchange rate in E^rns^ΔNΔC and should therefore in the truncated fragment be located at the membrane surface where it is exposed to water. Interestingly, the NH side group of Trp203 did not show a change in its water accessibility upon truncation, but is still located in a hydrophobic environment. Most notably, the water accessibility of the C-terminal amino acids is increased in E^rns^ΔNΔC. The peptide NH group of the four C-terminal amino acids Glu216, Asn217, Lys218 and Ser219 all exhibit an enhanced proton exchange rate once the last six amino acids are deleted. Moreover, the Thr202 NH group in E^rns^ΔNΔC shows a proton exchange rate that is comparable to the values determined for the aforementioned four amino acids. Interestingly, the side chain NH of Asn217 showed nearly the same low proton exchange rate in both proteins. This indicates that only the peptide bond has changed its position in E^rns^ΔNΔC whereas the side chain is still protected from water.

The results of the water accessibility experiments with E^rns^ΔN and E^rns^ΔNΔC are summarized in **Fig. 6B**. It is obvious that the deletion of the last six amino acids results in a change in water accessibility of upstream sequences and increases the number of NH bonds that are engaged in fast exchange. This occurs especially in the formerly water shielded central region of the E^rns^ protein. Interestingly enough, the flanking amino acids Thr201 and Lys214 did not gain direct access to water, but the accessibility of the peptide NH groups increased at both sides of the formerly shielded domain upon C-terminal truncation. In both deletion constructs, there remains a continuous stretch of 8 amino acids showing no water accessibility, including the side chain of Gln207. This finding of a discontinuity in the protection pattern at residues 216–219 does not support the picture of a perfectly straight amphipathic helix in the C-terminal region.

### Structural model of the C-terminal region

**Figure 7:**
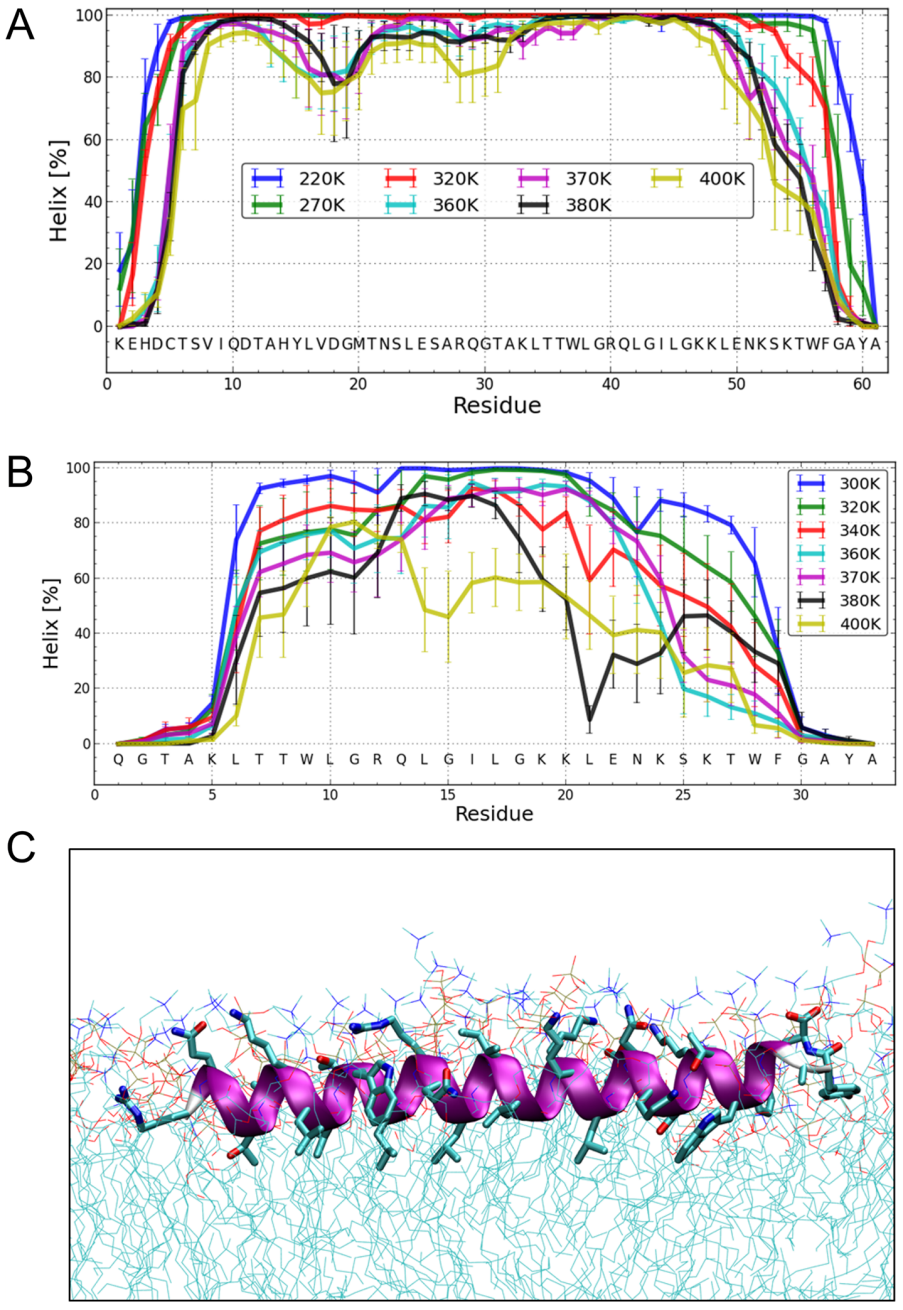
Structure simulations. (A) MC simulations in an implicit membrane model. Fraction of per-residue helical content at different simulation temperatures for E^rns^, as determined by the probability of finding a helical secondary structure element at the respective position. The value of the standard deviation is based on five trajectories per temperature. The simulations showed mostly helical conformations for about residues Thr172 to Lys218, whereas the terminal residues appear mostly disordered. (B) MC simulations in an implicit membrane model. Fraction of per-residue helical content at different simulation temperatures for E^rns^ΔN, as determined by the probability of finding a helical secondary structure element at the respective position. The value of the standard deviation is based on five trajectories per temperature. The simulations showed mostly helical conformations for residues Leu200 to Lys214, whereas the first five N-terminal residues are mostly disordered. With increasing temperature, the region between Leu215 and Phe223 partially unwinds and exhibits loop conformations followed by the helical C-terminus. (C) MD simulation of E^rns^ΔN (Arg194 – Ala227) at 72 ns in an explicit DMPC membrane. The protein shows a strong helical fold and lies slightly inclined in the hydrophobic region of the membrane just beneath the lipid head groups. The peptide was pulled from an equilibrated position in the membrane surface into the center of the membrane to mimic a more physiological starting position since at least part of the structure should be located within the membrane when the E^rns^ C-terminus is generated by signal peptidase cleavage of the E^rns^/E1 precursor. In a free MD simulation following the pulling, the peptide exhibited a kink around residue 202 (not shown) until 40 ns, then the peptide was back to the original surface bound orientation and full helicity.

To elucidate the degree of helicity of membrane-bound E^rns^ by an independent method, we performed all-atom Monte Carlo (MC) simulations with an all-atom intramolecular force field [41] that was recently used to describe the reversible folding of various proteins [42], [43]. To speed up the simulation, we employed an implicit membrane model with three layers providing discrete dielectric environments [44]. E^rns^ΔN (Fig. 7B) as well as the entire E^rns^ anchor (Fig. 7A) were simulated at temperatures ranging from 220K to 400K, starting from completely helical conformations that were placed in the proximity of the membrane surface. The region from Thr172 to Lys220 remained helical, while the N- and C-terminal regions showed partial unfolding with increasing temperature (see Supporting Information Fig. S4A). A similar picture emerged for E^rns^ΔN, as illustrated in Fig. 7B, which remained helical from Leu200 to Lys214. The last six C-terminal residues are unfolded for both peptides (Supporting Information Fig. S4A/C). Both peptides remained bound to the membrane surface in all simulations, with the hydrophobic residues facing inward. Moreover, the helix was slightly tilted (Supporting Information Fig. S5).

The Monte Carlo simulations can give a good prediction of the local secondary structure of E^rns^ΔN, but the implicit membrane model is not suitable to gain an impression of the depth of insertion or of the exact tilt angle of the protein within the membrane. We therefore conducted all-atom molecular dynamics (MD) simulations in an explicit lipid bilayer composed of 512 DMPC molecules, in order to obtain further information on the membrane insertion of E^rns^ΔN (Fig. 7C). The resulting MD model shows a pronounced helical conformation of E^rns^ΔN and a slightly tilted orientation in the membrane with an angle of 15° relative to the membrane surface. The peptide maintained a stable helical conformation throughout all simulations, but showed a kink around residue 202 when pulled into the membrane. Besides using the conventional MD approach of allowing the peptide to approach the membrane from the aqueous phase, the simulation in Fig. 7C was started from a position deep within the membrane. This should be a more relevant physiological starting position, since at least part of the structure should be located within the membrane when the C-terminus is generated by signal peptidase cleavage of the E/E1 precursor. The peptide maintained a stable helical conformation throughout the simulation, while the membrane initially bent to compensate for the peptide translocation when the protein was pulled into the membrane core. However, after 35 ns of free MD, the peptide was back to its original position near the membrane surface and assumed its stable orientation there.

## Discussion

E^rns^ represents one of the four known pestiviral structure proteins. The protein is crucial for the formation of infectious viral particles and plays a major role also during the infection of cells. But aside from this elementary function, E^rns^ represents also a virulence factor of pestiviruses. In this context it is important that E^rns^ has an intrinsic RNase activity. RNases are only very rarely found in RNA viruses [45], and to our knowledge the pestivirus E^rns^ is the only viral structural protein displaying such enzymatic activity. This RNase activity is crucial for the virulence of the virus. It could be shown that pestiviruses containing an RNase negative E^rns^ protein are viable and able to replicate to nearly wild type virus titers, but are attenuated in their natural hosts [21], [22]. Most importantly, the RNase activity is a major factor for establishing a persistent infection upon transplacental infection of the fetus in a pregnant host animal [4].

E^rns^ is not only concentrated within the cell at the site of virus budding, but it is also secreted to some extent from an infected cell and is thus found in the blood of infected animals [15], [16]. According to the working hypotheses put forward so far, the virulence factor activity of the E^rns^ RNase is supposedly linked directly to its secretion from infected cells. Accordingly, an analysis of E^rns^ membrane anchoring and the mechanisms underlying its partial secretion are of major importance for understanding its activity. It had been proposed earlier that E^rns^ should be bound to the host membranes and to the virion via interaction with the E2 protein that contains a typical transmembrane region [46]. Regardless of the question whether an E^rns^/E2 heterodimer is formed in all pestiviruses, the results presented here and data published before [24], [25], [26] clearly show that the E^rns^ C-terminus represents an intrinsic membrane anchor *per se*. The amphiphilic region serves to attach the protein to lipid bilayers and enables its intracellular retention in the absence of any other viral protein. Accordingly, the hypothesis of indirect membrane anchoring of E^rns^ achieved via interaction with E2 - as put forward by Lazar et al. [46] - is refuted by the data available now.

The structure of the E^rns^ N-terminal RNase domain was successfully determined by X-ray crystallography, yielding important clues to its enzymatic functions [20]. Unfortunately, the full-length E^rns^ protein containing its C-terminal membrane anchor region could not be crystallized, so that structural data on this functionally important domain are missing. We have therefore investigated the structure of the C-terminal domain using several complementary methods. First, we could demonstrate in cell culture experiments that this domain does indeed serve as the membrane anchor [24], [25]. However, in those experiments the membrane anchor did not bind to membranes as tightly as a typical transmembrane helix. Sequence prediction and model building showed that the E^rns^ membrane anchor could be structured as a long amphipathic helix. Mutations interfering with the proposed amphipathic character of the C-terminal region affected the membrane anchoring as well as the secretion of the protein [25], [26].

Peripheral membrane binding via amphipathic helices is quite common for both cellular and viral proteins. Cellular amphipathic helices are typically located at the N-terminus and consist of only 3–4 helical turns. Such proteins are found in the cytoplasm or the ER and have a broad range of important biological functions. For example, such structures are used in the cell for measuring membrane curvature [47], for generating membrane vesicles [48], [49], or for establishing protein-protein interactions [50]. The amphipathic helices are generally oriented parallel to the membrane surface and act, in the case of proteins involved in the budding of membrane vesicles, as a membrane anchor of these proteins. The local curvature of the membrane involved in vesicle budding is usually not induced by the amphipathic helix itself, but e.g. by the bent form of the proteins [49] or by protein-protein crowding [51].

The considerable length of the E^rns^ amphipathic helix (up to about 50 amino acids in TFE) is more reminiscent of amphipathic helices found in another group of proteins, such as cytolytic peptides. These peptides usually consist of an amphipathic helix composed of 23–31 amino acids [52]. Melittin, a toxic component of bee venom, for example, binds to cellular membranes and forms a membrane pore by oligomerization, which leads to the damage of the target cell. Similarly, the antimicrobial peptide PGLa is a straight amphiphilic α-helix, which binds flat to the membrane surface, whereupon it can start to tilt and eventually assemble transiently as an oligomeric transmembrane pore [34], [36], [38]. The melittin and PGLa helices can be slightly tilted into the membrane like the E^rns^ anchor [35], [37], [53], but the E^rns^ anchor most likely lacks the ability of a concentration depending structural re-orientation according to our experiments.

Several viral proteins are known to contain short amphipathic helices, such as the Brome-Mosaic-Virus protein 1a [54], or the NS3, NS4A, NS-4B and NS-5A proteins of hepatitis C virus [55], [56]. Also for pestiviruses membrane binding of NS5A was demonstrated to occur via an amphipathic helix similar to the closely related hepatitis C virus [57]. But all these systems represent non-structural proteins, and the amphipathic helix is only used for membrane attachment and oligomerization to build up the replication complexes on the cytoplasmic membrane surface [58]. To our knowledge E^rns^ is the only *structural* viral protein that is anchored via an amphipathic helix. This amphipathic helix is unusually long and located in the C-terminal region. It combines several functions, as it is not only important for the membrane binding of the protein, but also for the control of its secretion/retention, its intracellular location [26], and for cleavage of the glycoprotein E^rns^/E1precursor by the cellular signal peptidase [27]. All these different demands have to be supported by the specific conformation and particular sequence of the E^rns^ C-terminal region.

Our analyses revealed that the E^rns^ anchor is predominantly helical when bound to the membrane, but that the stability of the helix varies considerably over the sequence, with 15 residues representing a core helix that can be extended towards both sides. Monte Carlo (MC) simulations support the experimental data, as they yielded a similar extent of helicity and could identify the stretch Leu200 – Lys214 as the most stable helical region, in perfect agreement with CD and NMR data. Both the OCD analyses and the simulations revealed a slight tilt of the helix with respect to the membrane surface (Fig. 2F and Supporting Information Fig. S5, respectively).

Since the MC analyses do not provide information on the location of the anchor in the membrane, molecular dynamics (MD) simulations were conducted. The MD analyses also revealed a helical conformation with a slight tilt. The helix was, embedded just below the lipid headgroup region of the membrane. Unlike the other data, the helix in the MD simulations extended virtually over the complete length of the peptide. Thus, the helix content of this simulated structure is significantly higher than what was observed experimentally in the CD, NMR and MC analyses, which had shown unwinding of the N- and C-terminal residues. This discrepancy is most likely due to an overestimation of the H-bond energy in the force field.

As a general consensus of the CD, OCD, NMR, and MC simulation data, and in part also MD simulation results, at least part of the helix should be inserted more deeply in the membrane than the surrounding amino acids. This conclusion is in agreement with the data obtained by the NMR CLEANEX experiments showing that the central region of the amphipathic helix does not exchange the NH protons with water protons. Likewise, the side chain protons of Trp203 and Gln207 are protected and thus seem to be located in the hydrophobic interior of the bicelle. Nonetheless, the immersion within the bicelle is either not very deep or not perfectly stable, because all residues experience the influence of the paramagnetic agent gadolinium (Supporting Information Fig. S3). This ion induces strong relaxation that leads to a disappearance of the signals within a minimum radius of 5 Å [59]. The fact that the NMR signals of the full-length E^rns^ anchor show dynamic exchange most probably with the free unstructured protein also indicates that the interaction with the membrane-mimicking bicelles is only of moderate stability.

Interestingly, the N-terminal end of E^rns^ΔN is located on the bicelle surface or protrudes into the solvent, given its high water accessibility, while the amino acids of the far C-terminal end are shielded from water including the last C-terminal residue Ala227. Together with the observed NOE pattern, this result suggests that the C-terminal end of the sequence is at least temporarily located within the membrane, being quite flexible with regard to its secondary structure, as most clearly seen for Ala227, the very last C-terminal residue of the protein. Ala227 does not show any water contact, which argues for a membrane immersed location, but the relaxation and Het-NOE measurements revealed a flexible conformation. Any particular stable C-terminal conformation, such as a putative turn close to the water/lipid interphase was not reproduced in the MD simulations, possibly due to an overestimation of the helical hydrogen-bonding. However, the MC simulation revealed a significant probability for a turn-like structure at the C-terminus (Supporting Information Fig. S4D), and the same pattern was also revealed by MC simulations of the full-length E^rns^ anchor (Supporting Information Fig. S4B).

This model is supported by the pronounced influence that the native E^rns^ C-terminus has on the central helix, as seen from the increased water accessibility in the truncated construct E^rns^ΔNΔC. This effect was not restricted to the C-terminal region of the immersed part, but was also seen for residues located upstream at the N-terminal end of the inserted helix. Thus, the C-terminal end of the E^rns^ anchor has an effect on the immersion of the whole inserted segment. On the other hand, the N-terminal region of the protein did not show such an interesting effect, as all amide groups had strong water contacts.

The structural model of the E^rns^ C-terminus presented here does not answer all open questions about the biological mode of action of the E^rns^ anchor, because it does not allow any conclusions about the mechanism governing the equilibrium of E^rns^ retention and secretion. Nevertheless, it gives a first hint on how the interaction of the protein with the membrane occurs. Although the E^rns^ protein lacks a transmembrane domain or a GPI anchor, which are usually responsible for tight membrane association, its unusually long amphipathic helix confers strong membrane binding. This anchor is not only attached to the membrane via the hydrophobic face of the amphiphilic helix, but because of the tilt part of the helix including its far C-terminal end is inserted into the membrane. This may facilitate the arrangement of lipids around the inserted peptide and may lower the free energy of the system to stabilize the membrane/protein interaction. It thus appears feasible that the E^rns^ anchor could bind more strongly to membranes than a typical amphipathic helix on the bilayer surface, thereby providing the firm membrane association that is crucial for a viral surface protein. Nevertheless, the membrane affinity of this anchor is considerably lower than that conferred by a transmembrane helix [24], which seems to be an important prerequisite for the observed secretion of E^rns^. E^rns^ represents the first membrane protein for which anchoring via an amphipathic helix is described, and this unusual type of membrane attachment can be hypothesized to be of functional importance. The structure adopted by this anchor in a membrane could contribute to the known equilibrium between retention and secretion by adjusting the binding force at an appropriate level. This hypothesis is in agreement with the observation that C-terminally truncated E^rns^ is more efficiently secreted [24], [26], because the observed increased water accessibility of the central part of this truncated helix suggests that this region becomes less deeply immersed and consequently has a decreased binding affinity. Whether this proposed decrease of binding (alone) is responsible for increased secretion of the mutant protein cannot be decided yet. Alternatively, a different orientation of the protein in the membrane could interfere with the interaction between E^rns^ and other proteins important for its membrane association. Further investigation is necessary to answer the question whether other mutations enhancing secretion [25], [26] also alter the immersion and binding affinity of the helix. In a rather speculative working hypothesis, one might propose that either a slight truncation of the C-terminus or its unfolding as a result of (changing the) interaction with a partner molecule could destabilize lipid binding of the anchor and lead to secretion. So far, nobody has achieved a detailed analysis of the primary structure of the secreted protein, and any data concerning putative interaction partners of the anchor sequence are missing, so that both possibilities have to be investigated in future work. For the time being, the present data support a model that provides strong membrane binding, which, however, can be modulated by changes affecting the far C-terminal end or the overall structure of the anchor.

The structure model presented here displays the monomeric form of the protein but *in vivo*, a considerable amount of E^rns^ is found as a homodimer covalently linked via a disulfide bond between the Cys residues at position 171 of the protein [23], [60], [61], [62]. Nevertheless, we had no indication of dimer formation when analyzing the full-length anchor containing Cys171. The mass spectra of the purified protein did not reveal the presence of the dimeric form and the NMR analyses proved furthermore that every nucleus was found in only one defined surrounding. This observation implies that only one conformation - whether dimer or monomer - was present in the samples. For a dimer, this result could only be obtained in the case of ideal symmetry. Moreover, we conducted NMR analyses on the E^rns^ anchor both with and without the addition of DTT and were not able to identify any differences between these spectra (not shown). Most importantly, the NMR analyses leading to the structural model were conducted with a C-terminal part of the anchor lacking Cys171.Fraom biochemical analyses of the E^rns^ protein it is known that the ability to form dimers is massively weakened when the disulfide linkage is prevented by mutation of Cys171 [23]. Taken together, these results strongly support the notion that the E^rns^ anchor or fragments thereof analyzed in our experiments were monomers. E^rns^ dimerization is important *in vivo*, however, and the dimeric form is found in virions, infected cells, and also in the supernatant of infected cells. Knocking out E^rns^ dimer formation by mutation of Cys171 doesn't interfere with virus viability but leads to virus attenuation in the natural host [23], so dimer formation plays a role for E^rns^ function. Even though we do not have any data on the structure of a membrane anchored E^rns^ dimer, it is tempting to speculate on the structure of such molecule. We can conclude from the data on a peptide corresponding to the N-terminal part of the CSFV Alfort/Tübingen E^rns^ membrane anchor [26], as well as from the crystal structure of residues 1 to 165 of the BVDV NCP7 E^rns^ [20] that Cys171 is located in a rather flexible region of the protein, linking the enzymatically active N-terminal domain to the C-terminal membrane anchor. Hence, contact of the two monomers in this region should not be sterically hindered. In plane binding of the membrane anchor helices of both monomers parallel to the bilayer surface according to our structural model would also not interfere with protein/protein interaction between the regions containing Cys171. The angle between the two anchor helices in a two dimensional projection should be flexible, since there is no reason to postulate any particular fixed arrangement. Furthermore, as pointed out above, we have no indication that two or more anchor molecules could engage in a parallel or antiparallel alignment of the two amphipathic helices of an E^rns^ dimer. Whether the two regions around Cys171 of the two monomers form a parallel stem–like structure or whether they cross each other cannot be answered yet and also the membrane topology of this contact region is unclear at the moment. Further experimental work would be necessary to get an idea of the structure of the E^rns^ membrane anchor in a dimeric state. One important step towards elucidation of this point would be to identify residues that are part of the dimerization interface in the region around Cys171.

The model established here for the E^rns^ C-terminus describes only the folding of the fully processed protein. However, E^rns^ is not translated as a single protein but rather as a part of the pestivirus polyprotein that is posttranslationally cleaved into the different viral proteins. The N-terminus of E^rns^ is generated upon cleavage by the cellular signal peptidase [27], which is also responsible for several other steps of pestivirus polyprotein processing [15]. The signal peptidase usually cleaves after a so called von Heijne sequence that is preceded by a transmembrane helix [28], [29]. It has long been puzzling why and how the C-terminal end of E^rns^ can be generated by signal peptidase, given that this sequence lacks a transmembrane element [27]. The structural model for the E^rns^ C-terminus presented here cannot explain this conundrum, as we found no indication for a transmembrane orientation of the C-terminal segment. Therefore, the structure of the uncleaved E^rns^ C-terminus should differ from the final form of the processed C-terminus to allow the cleavage by the cellular signal peptidase. It seems likely that the E^rns^ C-terminus adopts an alternative transient structure during processing, while being tethered to the membrane via the transmembrane region of the E1 protein that follows downstream in the polyprotein. This sequence of events could explain why the cleavage between E^rns^ and E1 is delayed compared to other polyprotein processing steps [15].

Recently, a new structural motif named “charge zipper” was identified in the membrane binding domains of various proteins [30]. The contact between two amphipathic helices is stabilized through electrostatic interactions between charged amino acids on the two apposed segments, such that a long ladder of salt bridges is formed between them, either leading to an intramolecular hairpin, or resulting in intermolecular oligomerization. Such a charge zipper motif was also identified in the C-terminal membrane anchor of E^rns^. Folding of this region into a helical hairpin would allow a transmembrane insertion of the anchor, since the charged amino acids would be shielded within the structure, while hydrophobic residues would be present on the outer face and could be easily inserted into the membrane. The resulting structure could provide a (transient) transmembrane helical hairpin structure upstream of the E^rns^/E1 processing site, which might explain its cleavage by the signal peptidase. After cleavage, a structural rearrangement of the released E^rns^ C-terminus could occur, resulting in the identified structure of the mature E^rns^ C-terminus that allows membrane anchoring of E^rns^ and control of secretion. This charge zipper hypothesis is highly interesting but has to be analyzed in detail with further experiments.

The E^rns^ membrane anchor thus combines several very different functions in a rather short stretch of the sequence. These functions are important for the life cycle of pestiviruses. They rely on a specific structure that is probably established through different transition states, and which promotes membrane binding and signal peptidase processing. The final structure of the mature E^rns^ C-terminus presented here as a model describes for the first time a new way of anchoring a viral surface protein via an unusually long amphipathic helix to a membrane. This arrangement represents a new perception of protein/membrane interaction that might also be relevant for other peripheral membrane proteins. Importantly, the structural model of the E^rns^ C-terminus reveals not only a new possible fold of an amphipathic membrane anchor, but it also provides new insight into the biochemical requirements of the E^rns^ protein and paves the way towards a better understanding of the virulence function of this fascinating system.

## Materials and Methods

### Construction of plasmids

Plasmid pd29G was used as a starting point for all expression constructs. It consists of the plasmid pETZ2-1 (kindly provided by Gunter Stier [63]), which codes for a Z2 domain to enhance the water solubility of the fusion protein, a C-terminal His-TAG for protein purification and a N-terminal TEV protease cleavage site to allow removal of the complete TAG from the desired expression product. The insertion in pd29G consists of a cDNA coding for part of the viral polyprotein from BVDV strain CP7 E^rns^ (Lys167 - Ala227) after four cloning derived amino acids (GAMA). The cDNA sequence was optimized for the bacterial expression of the protein. Plasmid pd29G-1, containing the sequence information for the protein E^rns^ΔN (Arg194 - Ala227), was generated by a QuikChange (QC) PCR using ‘CCTGTATTTTCAGGGCCGTCAGGGGACCGC’ as forward and ‘GCGGTCCCCTGACGGCCCTGAAAATACAGG’ as reverse primer. QC PCR was conducted according to the protocol by Strategene (Heidelberg, Germany). The expression plasmid of E^rns^ΔNΔC (Arg194 – Thr221; plasmid pd29G-1-2) was established from pd29G-1 via QuikChange mutagenesis with primers ‘GAAAACAAAAGCAAAACCTAATAACTCGAGCACCACCACC’ and ‘GGTGGTGGTGCTCGAGTTATTAGGTTTTGCTTTTGTTTTC’. The established constructs were all verified by nucleotide sequencing with the BigDye terminator cycle sequencing Kit (PE Applied Biosystems, Weiterstadt, Germany). Sequence analysis and alignments were done with MultiAlin [64]. Cloning was done using standard procedures [65].

### Expression and purification of proteins

The expression of proteins was done with *E.coli* strain BL21(DE3) in standard LB-Medium for the CD measurement, or in minimal medium containing ^15^N (^15^NH_4_Cl, ISOTEC, Sigma-Aldrich, USA) and ^13^C (^13^C6 D-Glucose, Cambridge Isotope Laboratories, Inc, USA) for the NMR analysis. 1 l of medium was inoculated with an overnight culture until the OD_600_ of the mixture was between 0.05 and 0.1. The bacterial growth at 37°C and 220 rpm was observed until an OD_600_ of 0.8 was reached. At this point protein expression was induced by addition of IPTG (final concentration 0.5 mM). For expression in minimal medium, 2.5 ml 20% ^13^C6-Glucose per liter of culture was added to the medium. The bacteria were incubated at 20°C and 220 rpm and harvested after 3 h. After centrifugation for 10 min at 5000 g and 4°C the bacteria were resuspended in 15 ml *Lysisbuffer* [50 mM NaH_2_PO_4_ pH 8.0, 300 mM NaCl, 30 mM Imidazol, Lysozym, 6% TritonX-100, 1 tab. Roche Complete Protease inhibitor without EDTA (Roche, Mannheim, Germany)] per liter of minimal medium. After 10 min incubation at room temperature the bacteria were lysed by three freeze-thaw cycles performed with liquid nitrogen and warm water and sonification for 6×30 sec on ice (Branson Sonifier B15, level 7, cycle 80%). The insoluble debris was removed by centrifugation (Beckman JA17 rotor, 30 min, 31000 g) at 4°C.

The purification was started with a 5 ml Ni-NTA column (Protino Ni-NTA Columns, Macherey-Nagel, Germany) on an FPLC system (LKB GradiFrac, Pharmacia Biotech, Freiburg) with a flow rate of 3 ml/min. The UV absorbance at 280 nm was measured with a connected absorbance recorder (LKB Optical Unit, LKB REC102, Pharmacia Biotech, Freiburg) to identify protein containing fractions. A step gradient of 50 mM and 100 mM imidazole was used to prevent unspecific protein binding, and elution was accomplished with 300 mM imidazole. Afterwards, the protein containing fractions were pooled and ultrafiltrated (Amicon Ultra-15, Millipore). The retentate was diluted in *TEV-Buffer* (Invitrogen, USA) and again ultrafiltrated until the NaCl concentration was less than 5 mM. The protein concentration was determined by measuring the absorbance at 280 nm to calculate the amount of AcTEV-Protease (Invitrogen, USA) that was needed to cleave off the N-terminal Z2-TAG. It was assumed that 10 U AcTEV-Protease could cleave 2.16 mg substrate. To prevent oxidation, the solution was overlayed with CO_2_ and incubated for several days at room temperature. Each day a 1 µl sample of the solution was collected and analyzed to check the cleavage process. After cleavage was completed, the cleavage products were separated with a reverse phase HPLC (BT9200 Titan HPLC pump, Eppendorf, Hamburg) using a C4-Reprosil 300 column (Dr. Maisch GmbH, Ammerbuch-Entringen, Germany) and a gradient from 20% to 60% acetonitrile with 0.05% trifluoroacetic acid. The absorbance at 280 nm was recorded (BT9520 IN UV/Vis detector, LKB REC101, Pharmacia Biotech, Freiburg) and the eluent was collected in 2 ml fractions (Foxy Jr., ISCO) and analyzed. Positive fractions were pooled and lyophilized for several days. Afterwards, the molecular mass of the protein was measured using a MALDI-MS (Ultraflex I, Bruker) to determine the achieved isotope labeling, the purity and the absence of oxidation products.

### Circular dichroism (CD) spectroscopy

Phosphate buffer (PB), 2,2,2-trifluoroethanol (TFE) and sodium dodecyl sulfate (SDS) were obtained from VWR (Darmstadt, Germany). The detergent *n*-dodecylphosphocholine (DPC) and the phospholipids DMPC 1,2-dimyristoyl-*sn*-glycero-3-phosphatidylcholine (DMPC), 1,2-dimyristoyl-*sn*-glycero-3-phosphatidylglycerol (DMPG) and 1,2-dihexanoyl-*sn*-glycero-3-phosphatidylcholine (DHPC) used for vesicle and bicelle preparation were purchased from Avanti Polar Lipids (Alabaster, AL, USA). A weighed amount of lyophilized E^rns^ protein was dissolved in deionized water for preparing a 50 µM stock solution. SDS and DPC were used in a concentration of about 10 mM in 10 mM PB pH 6.5 with a protein/detergent ratio of 1∶600. The lipid powders of DMPC and DMPG were dissolved in 50∶50 chloroform/methanol (v/v) to get lipid stock solutions of ∼7 mM. Aliquots of these stock solutions were mixed in a glass vial and thoroughly vortexed to obtain the DMPC/DMPG mixture (1∶1 molar ratio). Subsequently, the organic solvent was removed under a gentle stream of nitrogen, followed by overnight incubation under vacuum. The DMPC or DMPC/DMPG lipid film that had formed in the vial was dispersed by the addition of 200 µl PB and homogenized by vigorous vortexing for 7×1 min and by 7 freeze-thaw cycles. Afterwards, small unilamellar vesicles were formed by sonication of the multilamellar vesicles for 4 min in a strong ultrasonic bath (UTR 200, Hielscher, Germany). The sonication procedure was repeated 3 times (with intermittent cooling of the water in the ultrasonic bath to room temperature with ice, to avoid overheating the samples). To prepare bicelles, a weighed amount of DHPC was first dissolved in 10 mM PB by sonification. An aliquot of this solution was used to dissolve a weighed amount of DMPC. Afterwards, the bicelle dispersion was homogenized by vortexing and freeze-thaw cycles as described above. Due to the significant technical challenges of examining bicelles in optical spectroscopy, which are caused by much stronger light dispersion compared to sonicated small unilamellar vesicles, the protein concentration of the sample was calculated by the UV-VIS absorption of a stock solution, from which the corresponding dilution factor could be determined.

To prepare the samples for CD analysis, an aliquot of the E^rns^ protein stock solution was added to PB, to a 50∶50 mixture of TFE/PB (v/v), to SDS or DPC micelles, or to the corresponding lipid dispersions. The final protein concentration in PB, TFE/PB and in micellar environment was 15 µM. In the lipid vesicle samples the protein concentration was adjusted in the range from 13–27 µM and the lipid concentration between 0.7–1.8 mM, resulting in peptide-to-lipid (P/L) ratios of ∼1∶20, 1∶50, 1∶100 and 1∶200. A 20 µM protein and 2 mM (total) lipid concentration and a P/L ratio of 1∶100 was set up in the bicelle samples by dissolving the lyophilized E^rns^ΔN protein directly in the bicelle dispersion.

CD spectra were recorded on a J-815 spectropolarimeter (Jasco, Groß-Umstadt, Germany) in rectangular quartz glass cells of 1-mm path length (Suprasil; Hellma, Müllheim, Germany) between 260 and 185 nm at 0.1-nm intervals. The temperature was set to 25°C for the peptide solutions in PB, the 50% TFE mixture, and the micellar solutions, and at 30°C for the vesicle or bicelle suspensions (*i.e.*, well above the lipid phase transition temperature of 23°C for DMPC and DMPG) using a water thermostat-regulated cell holder. Three repeat scans at a scan rate of 10 nm/min, 8 s response time, and 1 nm bandwidth were averaged for each sample and for the baseline of the respective peptide-free sample. After subtracting the baseline spectra from the sample spectra, CD data were processed with the adaptive smoothing method in the Jasco Spectra Analysis software. To calculate the mean residue ellipticities required for quantitative secondary structure estimation, the concentration of the peptide stock solutions was determined from the UV absorbance of the respective peptide at 280 nm. For better comparison of the spectra of the different samples, the calculated mean residue ellipticity (MRE) is shown in the graphs.

Secondary structure analyses were performed using the CDSSTR program [66], [67] with the implemented singular value decomposition (SVD) algorithm; by the CONTIN-LL [68], [69] program, which is based on the ridge regression algorithm; and by the SELCON-3 [70], [71] program, which incorporates the self-consistent method together with the SVD algorithm to assign protein secondary structure. The three algorithms are provided by the DICHROWEB online server [72], [73]. The quality of the fit between experimental and back-calculated spectrum according to the secondary structure fractions was assessed from the normalized root mean square deviation (NRMSD), with a value <0.1 considered as a good fit [72].

### Oriented circular dichroism (OCD) spectroscopy

Oriented protein-lipid samples for OCD measurements were prepared by depositing the proteolipid vesicles (with DMPC and DMPC/DMPG, as described above) on a planar quartz glass substrate. Each sample was generated by spotting a 60–80 µl aliquot of the vesicle sample onto a 20 mm diameter quartz glass plate (SUPRASIL, Hellma, Jena) as a ∼12 mm central circular spot, and dried under a gentle stream of air. Afterwards, the sample was rehydrated for 15 h at 30°C and 97% relative humidity in an OCD sample cell using a reservoir of saturated K_2_SO_4_ solution. The in-house built OCD cell can be integrated in a J-810 spectropolarimeter as an accessory, and further details on the OCD sample preparation and measurements have been described [33], [74]. The thin oriented bilayers formed during hydration of the sample minimize the possibility of undesired spectral artifacts caused by linear dichroism or absorption flattening. The OCD spectra were recorded as an average of 8 scans with a 45° rotation of the cell after each scan to further reduce spectral artifacts due to linear dichroism arising from imperfections in the sample, strain in the quartz glass windows, or imperfect alignment of the window. For OCD measurements the same data acquisition parameters were used as in the conventional CD experiments described above. Background spectra of pure lipid bilayers (without protein) were subtracted from all OCD spectra. In order to compare the different OCD spectra in a better way all spectra were normalized to match their ellipticity around the minimum at ∼220 nm.

### NMR spectroscopy

The NMR analysis was carried out in a lipid bicelle system with a total lipid concentration of 200 mM in PBS (50 mM KH_2_PO_4_ pH 6.8, 50 mM NaCl), using DHPC and DMPC (Avanti Polar Lipids, Alabaster, AL, USA) at a ratio of 4∶1. For sample preparation, the weighed amount of DH/MPC was dissolved in 450 µl PBS by vortexing and sonification until the solution was clear. Afterwards, the required amount of DH/MPC was added and the mixture was again sonified. Thereafter, 0.5 µmol of the lyophilized protein and 50 µl D_2_O were added. After another round of sonification the insoluble material was removed by centrifugation for 20 sec. at 320 rcf to get a clear solution. The protein/lipid ratio obtained by this procedure was 1∶222.

NMR experiments were performed on a Bruker Avance I 600 MHz spectrometer with a broadband triple resonance probe, or on a Bruker Avance III 600 MHz spectrometer with a cryo probe and a Z-gradient.

The ^15^N-HSQC and CLEANEX experiments [40], and titration with Gd 1,4,7,10-tetraazacyclododecane-1,4,7,10-tetraacetic acid (Gd-DOTA, Sigma-Aldrich) were typically acquired with 2- 4 scans and a total of 128 increments in the indirect dimension, between 23 and 50°C. Titration with Gd-DOTA was performed on 0.5 mM sample of E^rns^ΔN in either DHPC/DMPC or DPC micelles. A CLEANEX spin-lock field of 4.8 kHz was applied for a mixing time of 100 ms. 3D ^15^N-HSQC NOESY and ^15^N-HSQC-TOCSY experiments were performed at 23°C with a mixing time of 120 ms (NOESY) and 60 ms (TOCSY). 200–250 increments in the ^1^H dimension and 55 increments in the ^15^N dimension were acquired. A 3D ^13^C-HMQC NOESY was acquired in 200 mM deuterated DPC (Avanti Polar Lipids, Alabaster, AL, USA). ^1^H ^15^N ^13^C triple resonance experiments (HNCACB, HNCA, CBCA(CO)NH) at 23°C [75] were used to assign the ^1^H_N_, ^13^C and ^15^N resonances. ^15^N longitudinal (R1) and transversal (R2) relaxation as well as the heteronuclear NOE (het-NOE) were measured at 27°C. Relaxation delays varied between 10.8 and 3466.8 ms for R1, and 14.4 to 259.2 ms for R2 [76]. One duplicate point was included to test for instabilities. The heteronuclear NOE was determined as the signal intensity ratio of ^1^H_N_/N crosspeaks with and without ^1^H saturation. All experiments were recorded in an interleaved manner [77]. The water signal was suppressed with a combination of the water-flip-back and the WATERGATE scheme in all cases.

Spectra were processed using the nmrPipe software package [78] and analyzed with NMRView [79]. The normalized proton exchange rate was calculated by the intensity of the peak in the CLEANEX spectra divided by the intensity of the correlated peak in the ^15^N-HSQC spectra

### Monte Carlo simulations

Using the Monte Carlo simulation package SIMONA [80] we performed a total of 1.2×10^9^ steps, each of which changed a single dihedral, for both E^rns^ systems in the all-atom AMBER99SB-ILDN force field [41], in combination with an accurate implicit description of the solvent and membrane interactions [44]. Starting from an ideal all-helical conformation in the proximity of the outermost membrane layer, all simulations showed a quick attachment to the membrane surface. Depending on the simulation temperature (E^rns^ - 220K, 270K, 320K, 360K, 370K, 380K, 400K; E^rns^ΔN - 300K, 320K, 340K, 360K, 370K, 380K, 400K), the secondary structure varies over the sequence. We determined the standard deviation for the per-residue secondary structure information from five distinct populations per temperature, using the secondary structure assignment package DSSP [81]. For each population we averaged the fraction of secondary structure elements over every 10,000^th^ step, neglecting the initial 8×10^5^ steps to allow for equilibration.

### Molecular dynamics simulations of E^rns^ΔN

MD simulations were conducted using the molecular simulation package GROMACS 4.5.5 [82]. The AMBER99SB-ILDN force field [41] was used for the peptide, together with the SLIPID force field for the DMPC bilayer. E^rns^ΔN (Arg194 – Ala227) was constructed as an ideal helix with an acetylated N-terminus using the program xleap from the AmberTools package [83]. The peptide membrane complex was formed during an unrestrained membrane binding simulation of 10 ns length, by placing the peptide molecule parallel to a pre-equilibrated lipid bilayer at a distance of 1.8 nm above the lipid headgroups, at an elevated temperature of 480 K to speed up insertion. During the binding simulation, hydrogen bonds within the peptide were restrained to prevent unfolding. After cooling down, the system was equilibrated at 303 K with position restraints of 1000 kJ/(mol nm^2^) on the peptide for 500 ps. After this, an unrestrained MD simulation of 500 ns length was conducted using a Nose-Hover thermostat [84] and Parrinello-Rahman barostat [85], with semiisotropic pressure coupling. A time step of 2 fs was used together with the LINCS algorithm [86] to constrain bonds involving hydrogen atoms. Long range electrostatics were treated via PME combined with a 1.4 nm direct space cutoff for vdW and Coulomb interactions.

For another set of pulling simulations, the GROMACS pull code was used. The peptide was pulled into the membrane with a force constant of 10000 kJ/(mol nm^2^) and a pull rate of 0.2 pm/ps along the membrane normal for 10 ns. Then, the system with the peptide in the center of the bilayer was equilibrated for 500 ps using position restraints of 1000 kJ/(mol nm^2^) on the peptide. After that, another unrestrained MD simulation of 75 ns length was conducted.

## Supporting Information

Figure S1 Illustration of the characteristic OCD line shapes of an α-helical peptide for transmembrane (dotted line), tilted (solid black line) or parallel (dashed line) orientation with respect to the membrane surface. Theoretical OCD spectra have been calculated according to the method described in [87]. If the helix adopts an alignment parallel to the membrane surface the minimum at 208 nm has a stronger negative intensity than the minimum at ∼225 nm, if the helix is tilted the intensity of the 208-nm band is lower compared with the intensity of the 225-nm band, and if the helix is oriented transmembrane, the 208-nm negative band completely vanishes and intensity is close to zero or even slightly positive.
(PPTX)

Figure S2 Comparison of ^1^H^15^N-HSQC spectra of the full length E^rns^ anchor (A) and the N-terminally truncated E^rns^ΔN (Arg194 – Ala227) (B) in DPC micelles. Although the full-length E^rns^ anchor yielded good quality ^1^H^15^N-HSQC spectra, the corresponding 3D-^15^N-HSQC-NOESY and 3D-^15^N-TOCSY spectra (C) suffered from line broadening that impeded sequential assignment and showed a lot of empty strips.
(PPTX)

Figure S3 Effect of paramagnetic relaxation enhancement on the intensity of the peaks in ^1^H-^15^N-HSQC spectra of E^rns^ΔN. 0.25 mM Gd-DOTA were added to 0.5 mM E^rns^ΔN in 200 mM DPC, 20 mM KPI, 50 mM NaCl, pH 6.8 at 26°C. I, I0 - Intensities in the absence and presence of Gd-DOTA.
(PPTX)

Figure S4 The per-residue amount of structural turn (A, C) and coil (B,D) elements for each simulation temperature of E^rns^ (A,B) or E^rn^sΔN (C,D) was determined by the probability of finding the secondary structure element at the respective sequential position, averaged over every 10,000th step of distinct Monte Carlo simulations. The value of the standard deviation is based on five trajectories per temperature starting from an initially complete helical structure.
(PPTX)

Figure S5 Calculation of the tilt angle of E^rns^ΔN with respect to the membrane surface. The tilt angle was determined by initially guessing the helix axis based on the eigenvector of the peptide's inertia tensor, followed by an iterative optimization to find the optimal helix orientation. For this purpose, only the helical region spanning from residue Lys200 to Lys214 was considered. At each temperature, the tilt angle was calculated for five distinct populations, where every 10,000th snapshot was taken into account. The plot shows a pronounced tilt angle between 8 and 14 degrees with respect to the membrane surface, where the C-terminus points away from the membrane.
(PPTX)
